# Does mindfulness mediate the relationship between anxiety and second language performance? A meta-analytic structural equation modeling study

**DOI:** 10.3389/fpsyg.2026.1921078

**Published:** 2026-08-14

**Authors:** Yang Song, Yanxuan Chen, Junfeng Huang

**Affiliations:** College of Liberal Arts, Hubei University of Education, Wuhan, China

**Keywords:** anxiety, meta-analysis, mindfulness, second language performance, structural equation modeling

## Abstract

**Background and aims:**

Anxiety is a well-established affective factor that impairs second language (L2) learning, whereas mindfulness may alleviate anxiety and enhance cognitive processing. However, whether mindfulness serves as a mechanism linking anxiety to L2 performance remains unclear. This study aimed to investigate the mediating role of mindfulness in the relationship between anxiety and L2 performance.

**Methods:**

A systematic review was conducted for studies published up to April 2026. Data were extracted from observational studies (Pearson's correlations) and from baseline scores of intervention studies. Random-effects univariate meta-analyses were performed for each bivariate relation, and meta-analytic structural equation modeling (MASEM) was applied to test the mediation model. Moderator analyses were conducted using subgroup comparisons.

**Results:**

The meta-analysis included 13 studies (17 effect sizes) for the anxiety-mindfulness relation, 11 studies (14 effect sizes) for mindfulness-L2 performance, and 35 studies (41 effect sizes) for anxiety-L2 performance. Univariate meta-analyses showed that anxiety was negatively correlated with mindfulness [*r* = −0.32, 95% CI (−0.36, −0.27)] and with L2 performance [*r* = −0.34, 95% CI (−0.38, −0.30)], while mindfulness was positively correlated with L2 performance [*r* = 0.20, 95% CI (0.10, 0.28)]. MASEM revealed a saturated model with the following standardized path coefficients: anxiety → mindfulness β = −0.32 (*p* < 0.001), mindfulness → L2 performance β = 0.11 (*p* < 0.001), and anxiety → L2 performance (direct effect) β = −0.30 (*p* < 0.001). The indirect effect of anxiety on L2 performance through mindfulness was β = −0.03 (*p* < 0.001), and the total effect was β = −0.34 (*p* < 0.001).

**Conclusions:**

Mindfulness partially mediates the anxiety-L2 performance relationship, although the indirect effect is very small (β = −0.03). Anxiety is directly associated with lower L2 performance and also indirectly related to it via reduced mindfulness. Because this estimate was derived from pooled correlations across studies, it reflects an aggregate mediation pattern rather than a direct within-sample observation, and thus warrants cautious interpretation. Future research should explore additional mechanisms and use longitudinal designs to establish causality.

**Systematic review registration:**

https://www.crd.york.ac.uk/PROSPERO/view/CRD420261377122, identifier: CRD420261377122.

## 1 Introduction

Foreign language anxiety (FLA) is conceptualized as a distinct psychological construct that is significantly negatively correlated with academic achievement, classroom participation, and learning motivation (Aida, 1994; MacIntyre and Gardner, 1994). Extensive research in second language acquisition has demonstrated that anxiety not only affects attention, working memory, classroom engagement, and the quality of language output but also indirectly impairs language performance by reducing willingness to communicate and increasing avoidance behaviors (MacIntyre, 2017). However, despite the well-established detrimental effects of anxiety on language learning outcomes, the specific psychological mechanisms through which anxiety influences learning results remain insufficiently explained. In recent years, research has gradually conceptualized language anxiety as a factor that disrupts learners' self-regulatory functions (MacIntyre and Gardner, 1994). Attentional control theory (Eysenck et al., 2007) provides a detailed mechanistic explanation for this process. Anxiety impairs the efficiency of the central executive system, reducing learners' ability to allocate attentional resources to task-relevant information while increasing attention to task-irrelevant stimuli. In the context of L2 learning, this means that anxious learners are more likely to worry about their performance, fear negative evaluation, and engage in self-deprecating thoughts, thereby consuming cognitive resources that could otherwise be devoted to language processing and production (MacIntyre and Gardner, 1994). Mindfulness is defined as the awareness that emerges through paying attention on purpose, in the present moment, and non-judgmentally (Kabat-Zinn, 2003). According to Monitor and Acceptance Theory (MAT) (Lindsay and Creswell, 2019), the two core components of mindfulness-monitoring of present-moment experience and non-reactive acceptance-work synergistically. In the context of L2 learning anxiety, non-reactive awareness is particularly critical: it enables learners to observe their anxious thoughts without being entangled by them, thereby interrupting the automatic negative association between anxiety and performance and maintaining attentional stabilization and cognitive decentering. From this perspective, mindfulness is not only a positive psychological trait but also a self-regulatory mechanism that may buffer anxiety-related cognitive dysfunction.

The relationship between mindfulness and L2 performance has attracted relatively less attention than the anxiety–performance link, but available evidence is generally positive. Trait mindfulness—measured by instruments such as the Mindful Attention Awareness Scale (Brown and Ryan, 2003) and the Five Facet Mindfulness Questionnaire (FFMQ; Baer et al., 2006—has been associated with higher L2 speaking performance (Sipahutar, 2020), better vocabulary retention (Zeilhofer and Sasao, 2022), stronger reading comprehension (Alsharhani et al., 2023), and higher self-rated proficiency (Gao, 2023; Ulivia et al., 2022). Mechanistically, it has been proposed that mindfulness enhances L2 performance through two routes: by reducing test anxiety in high-stakes situations (Morgan and Katz, 2021), and by strengthening the metacognitive awareness and deliberate attention allocation required for language processing tasks (Fathi and Aghamirzaei, 2026). Nonetheless, effect sizes reported in individual studies vary considerably, and no meta-analysis has yet synthesized this specific relationship, leaving the overall magnitude of the mindfulness–L2 performance association uncertain. Although extensive research has established direct links between anxiety and L2 performance (Teimouri et al., 2019) and between mindfulness and L2 performance (Zhang and Zheng, 2026), whether mindfulness serves as a stable psychological mechanism linking anxiety to L2 performance remains lacking systematic theoretical and statistical validation. Several theoretical frameworks suggest that mindfulness may partially explain how anxiety affects L2 performance, and empirical studies have provided preliminary support for this mediation model. Fallah (2016) found that trait mindfulness negatively predicted L2 anxiety, which in turn positively predicted L2 achievement. Similarly, Shen (2022) reported that mindfulness reduced foreign language classroom anxiety, linking to better listening comprehension. However, other studies have found inconsistent results, with some showing no indirect effect of mindfulness on L2 performance via anxiety (e.g., Zhang and Zheng, 2026). Given these conflicting findings, it remains unclear whether mindfulness consistently serves as a self-regulatory pathway through which anxiety affects L2 performance. Therefore, a meta-analytic synthesis is needed to integrate existing research findings and systematically evaluate the strength and consistency of the mediating role of mindfulness in the relationship between anxiety and L2 performance. This meta-analysis has two primary aims. The first aim is to conduct a correlational meta-analysis to estimate the overall pooled effect sizes among foreign language anxiety (FLA), trait mindfulness, and L2 performance, and to examine potential moderators of these relationships, namely age (adolescents vs. adults) and educational level (K-12/school vs. university). Based on relevant theoretical frameworks and existing empirical evidence, we hypothesize that FLA is negatively associated with both trait mindfulness and L2 performance, whereas trait mindfulness correlates positively with L2 performance. The second aim is to employ meta-analytic structural equation modeling (MASEM) to examine whether mindfulness mediates the association between FLA and L2 performance. Specifically, we hypothesize that FLA produces a significant indirect effect on L2 performance through mindfulness, indicating that mindfulness acts as a partial mediator in the FLA–L2 performance relationship.Clarifying these correlations and their moderating patterns helps elaborate how FLA affects L2 learning outcomes through self-regulatory processes and deepens our understanding of the situational constraints of mindfulness-related mechanisms across different learner groups.

## 2 Method

### 2.1 Search strategy

To locate relevant studies, four electronic databases were consulted: PsycINFO, CNKI, Pubmed, and Web of Science. The literature search was conducted in December 2024 and updated in April 2026 for peer-reviewed journal articles and doctoral dissertations. The search query included terms related to anxiety (e.g., “anxiety,” “foreign language anxiety,” “FLA,” “language anxiety”), mindfulness (e.g., “mindfulness,” “trait mindfulness,” “mindful attention,” “mindful awareness”), and second language performance (e.g., “second language,” “L2,” “language achievement,” “language proficiency,” “language learning”). Boolean operators were used to combine these terms. The search was limited to studies published in English. The detailed Search Strategy is shown as in supplementary Tables 1–4.

### 2.2 Inclusion and exclusion criteria

Studies were included if they met the following criteria: (1) published in peer-reviewed journals or doctoral dissertations; (2) written in English; (3) examined the association between anxiety and mindfulness, or between mindfulness and L2 performance, or between anxiety and L2 performance; (4) reported Pearson's correlation coefficients between variables of interest; (5) for intervention studies, provided baseline correlations before treatment. Studies were excluded if they were qualitative, review articles, or did not provide sufficient statistical information to compute effect sizes.

### 2.3 Data extraction

Pearson's correlation coefficient (r) was used as the measure of effect size. For each study, the following information was extracted: author(s), year of publication, sample size, participant characteristics (age, educational level), measures of anxiety, mindfulness, and L2 performance, and the correlation matrix among variables. When a study reported multiple effect sizes from independent samples, these were treated as separate entries. When a study reported multiple measures of the same construct (e.g., multiple anxiety scales), composite correlations were computed following established procedures.

### 2.4 Meta-analytic procedure

#### 2.4.1 Effect size calculations

The meta-analytic procedure adopted in this study consisted of five core steps (Assink and Wibbelink, 2016). First, correlation matrices and their corresponding sample size data were extracted from eligible primary studies. Second, Fisher's *z*-transformation was performed to convert raw correlation coefficients into normally distributed z-scores, eliminating the skewed distribution issue of correlation values. Third, the standard errors of the transformed Fisher's *z*-scores were calculated, and each effect size was weighted by the inverse of the squared standard error to prioritize more precise study estimates. Fourth, the weighted mean *z*-score was computed based on the assigned weights, which was then back-transformed into original correlation coefficients via the inverse Fisher's *z*-transformation to obtain pooled effect sizes.

Subsequently, exploratory subgroup analyses were conducted to detect potential heterogeneity in effect sizes across different conditions. The subgroup stratification was implemented according to the categorical variables: educational stage (e.g., secondary education vs. higher education), L2 Skill Domain, Education level, Language Type, Study Region. Notably, these subgroup analyses were exploratory rather than confirmatory, as no *a priori* hypotheses were proposed regarding the differentiated effects of distinct negative emotion types. Publication bias was assessed using funnel plots (Doucouliagos et al., 2014).

Finally, the synthesized pooled correlation matrix was used as the fundamental input data for Meta-Analytic Structural Equation Modeling (MASEM). This standardized and rigorous analytical workflow guaranteed the accuracy of effect size estimation and enhanced the comparability of results across relevant studies. The specific formulas for Fisher's *z*-transformation, weighted average *z*-score calculation, and pooled correlation coefficient back-transformation are specified as follows:

Fisher's *z*-transformation formula for single-study correlation coefficient is shown in Equation (1)


$$\begin{array}{l} z_{x}=\frac{1}{2}\ln\left(\frac{1+r_{x}}{1-r_{x}}\right) \end{array}$$ (1)

where *r*_*x*_ refers to the raw correlation coefficient of the *x*-th independent study, and *z*_*x*_ denotes the corresponding standardized Fisher's *z*-score after transformation.

**Weighted average Fisher's z-score formula across all studies is presented in** **Equation 2:**


$$\begin{array}{l} z_{r}=\frac{\sum_{x=1}^{k}\left(n_{x}-3\right)z_{x}}{\sum_{x=1}^{k}n_{x}} \end{array}$$ (2)

where *z*_*r*_ represents the overall weighted average z-score synthesized from all included studies; *n*_*x*_ is the sample size of the *x*-th study; and *k* stands for the total number of independent effect sizes included in the meta-analysis in Equation 3. Inverse transformation formula for pooled correlation coefficient where *r* is the final pooled correlation coefficient obtained after inverse transformation of the weighted average *z*-score, which serves as the comprehensive effect size of the meta-analysis.


$$\begin{array}{l} r=\frac{e^{2z_{r}}-1}{e^{2z_{r}}+1} \end{array}$$ (3)

Heterogeneity across studies was assessed using both the *Q*-test and the I^2^ statistic. The Q-test follows a chi-square distribution with k−1 degrees of freedom (where k is the number of effect sizes). Significant heterogeneity was considered present when the *Q*-test yielded a *p*-value <0.05 and the I^2^ value exceeded 60%. Under such circumstances, a random-effects model was adopted; otherwise, a fixed-effects model was applied. All statistical analyses were performed using Stata (StataCorp LP), which automatically computes the Q-statistic and provides pooled effect size estimates for both modeling approaches.

#### 2.4.2 Meta-analytic structural equation modeling (MASEM)

**Figure 1:**
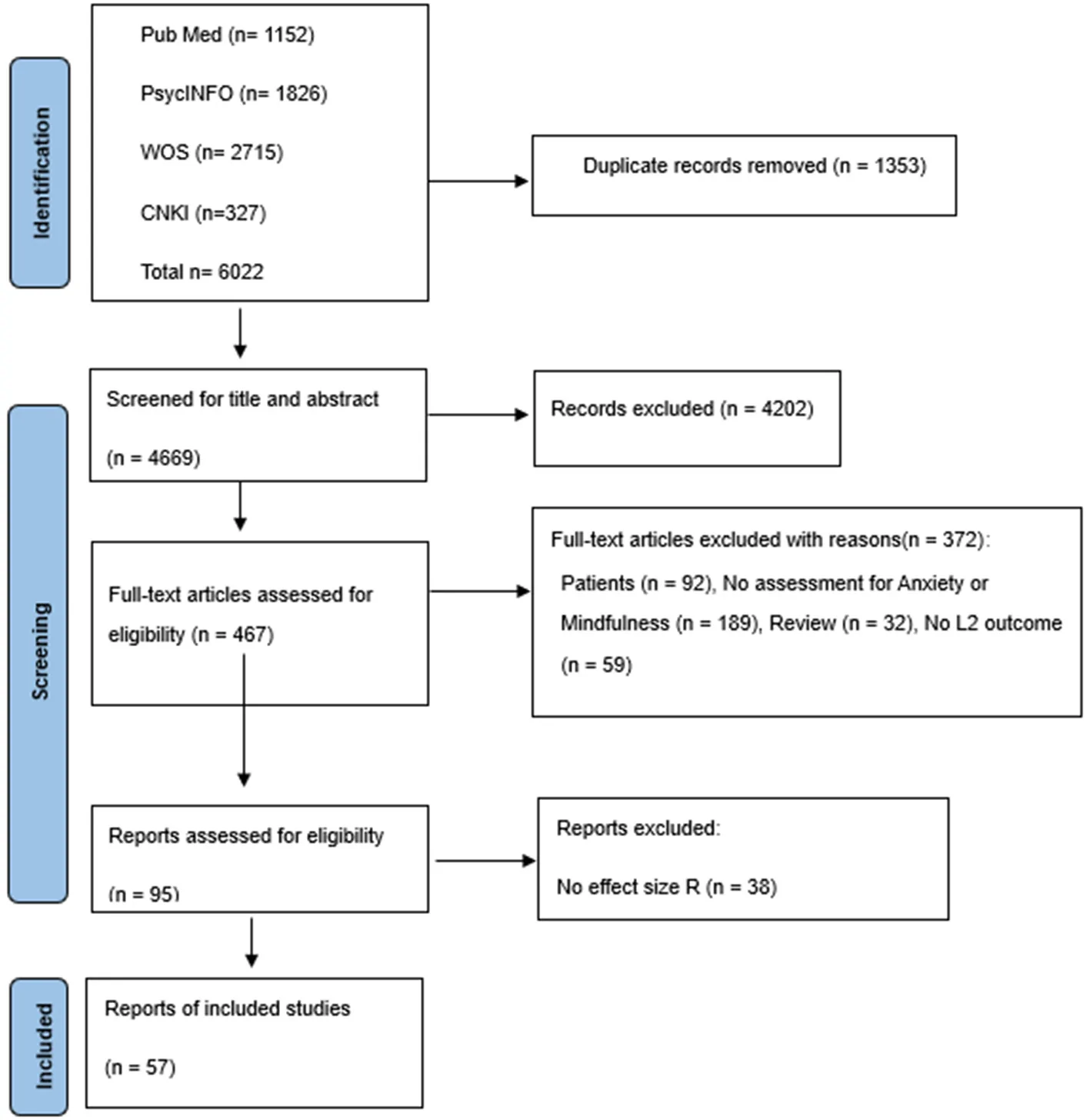
PRISMA flow chart of included studies.

To test whether mindfulness mediated the relationship between anxiety and L2 performance, a random-effects MASEM was applied (Jak et al., 2021). This approach synthesizes correlation matrices from individual studies to evaluate a common structural model. The mediation model (Figure 1) specified anxiety as the independent variable (X), mindfulness as the mediator (M), and L2 performance as the dependent variable (Y). The indirect effect was calculated as the product of the path from anxiety to mindfulness (Path a) and the path from mindfulness to L2 performance (Path b). The total effect of anxiety on L2 performance was decomposed into direct and indirect components. Parameter estimates were obtained using full-information maximum likelihood estimation. The overall effect size for each relation was computed using the ‘metafor‘ package in R (Viechtbauer, 2010).

#### 2.4.3 Moderator analyses

Moderator analyses examined whether the effect sizes varied by age (adolescents vs. adults) and educational level (school vs. university). Categorical moderator variables were included in separate meta-analyses using the ‘metaSEM‘ package (Cheung, 2015). A categorical moderator was considered significant if the 95% confidence intervals for the subgroups did not overlap.

## 3 Results

### 3.1 Study selection and characteristics

The systematic search identified 6,022 records. After duplicate removal and screening, 13 independent studies contributed to the anxiety-mindfulness relation, 11 to the mindfulness-L2 performance relation, and 35 to the anxiety-L2 performance relation. The total number of unique independent studies after removing overlaps was 57(as shown in Figure 1). Detailed characteristics of each study are provided in the supplement 5–7. Based on the Newcastle-Ottawa Scale assessment of all studies, the overall methodological quality is predominantly moderate (58.6%), with 27.6% rated high and 13.8% low (as shown in Supplement Table 8 Summary of the Quality Assessment Table). High-quality studies are mostly experimental or longitudinal, featuring pre-test and follow-up assessments. Moderate-quality studies are mainly cross-sectional, using validated instruments but often lacking control for confounders and follow-up. Low-quality studies include qualitative or topic-irrelevant research. Key strengths include adequate sample sizes and reliable measures, while limitations include cross-sectional designs, potential common method bias, and insufficient confounder control. The evidence base is generally sound for meta-analysis, though caution is warranted when interpreting results from lower-quality studies. Sensitivity analyses excluding low-quality studies are recommended.

### 3.2 Multi-level meta-analysis

**Figure 2:**
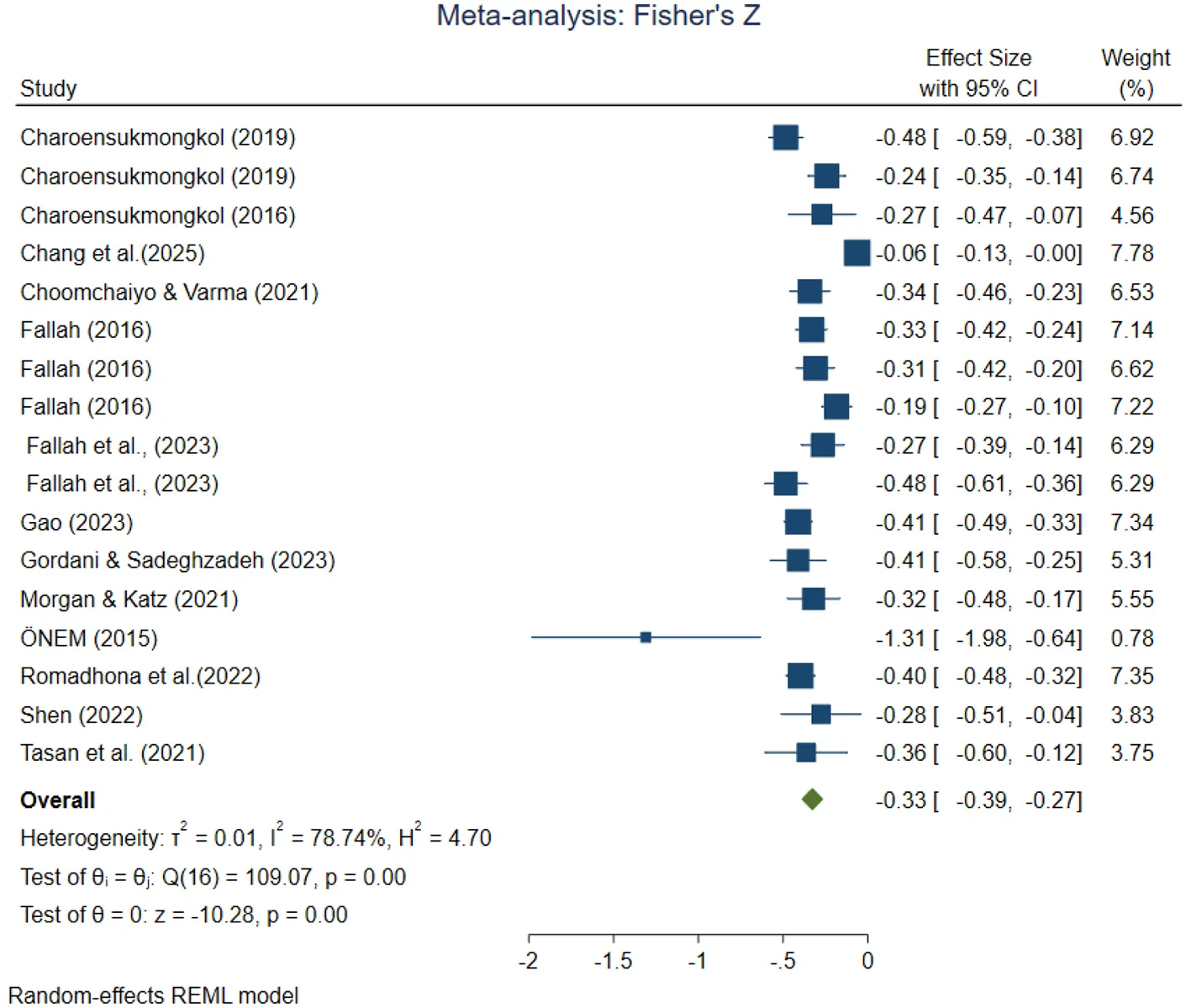
Forest plots of the association between anxiety and mindfulness.

Figure 2 presents the results of the separate meta-analyses for the relation between anxiety and mindfulness (path a-b). This meta-analysis included the following studies: Charoensukmongkol (2019), Charoensukmongkol (2016), Chang et al. (2024), Choomchaiyo and Parvathy (2021), Fallah (2016), Fallah et al. (2023), Gao (2023), Gordani and Sadeghzadeh (2023), Morgan and Katz (2021), Önem (2015), Romadhona et al. (2022), Shen (2022), and Tasan et al. (2021). A random-effects meta-analysis was conducted for each path. The weighted average correlation was r = −0.32 (Fisher's *z* = −0.339) based on 13 independent studies providing 17 effect sizes, with high heterogeneity (*I*^2^ = 78.74%, *p* < 0.001) and a significant overall effect (*z* = −10.28, *p* < 0.001).

**Figure 3:**
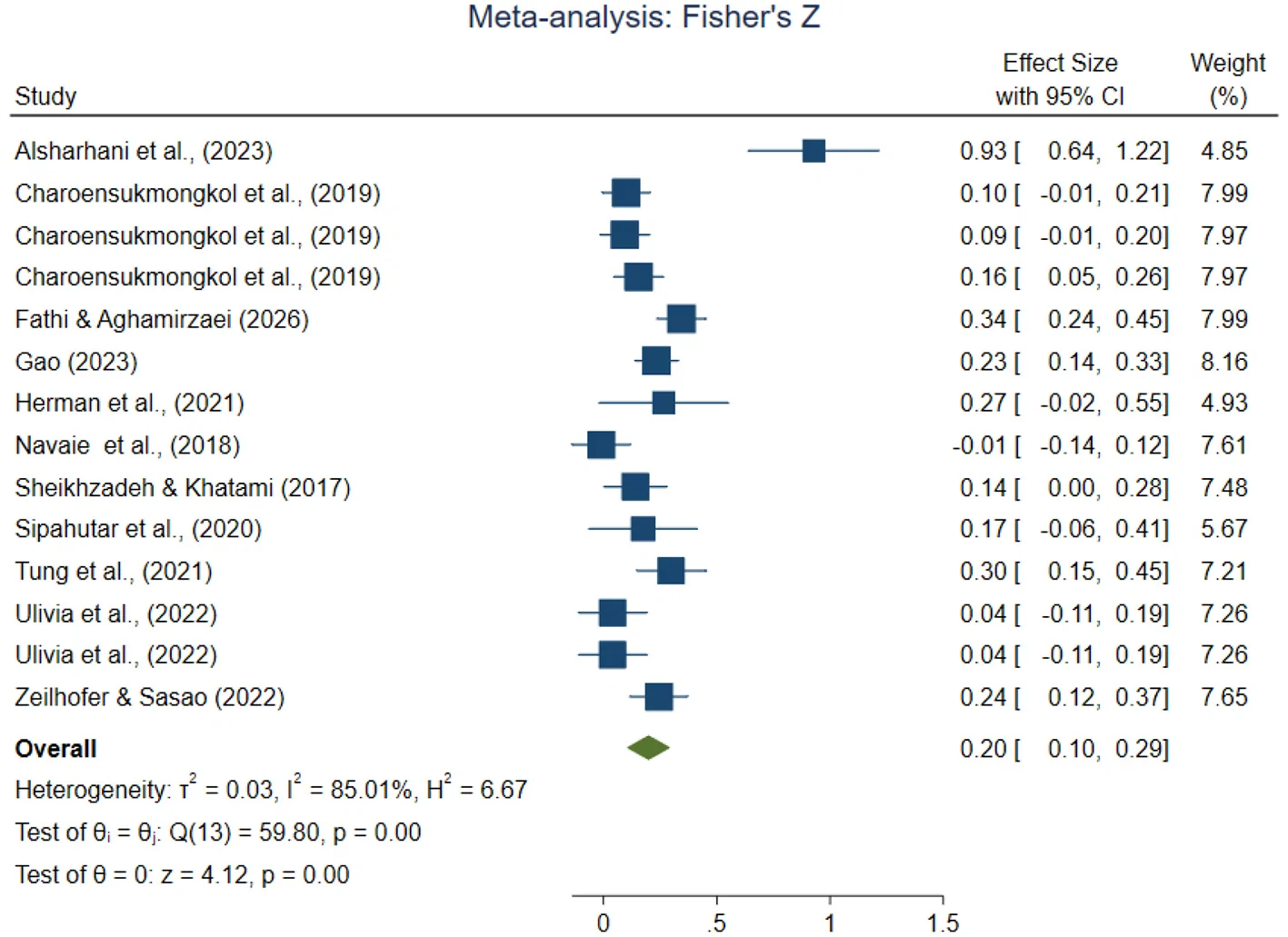
Forest plots of the association between mindfulness and L2 performance.

For the relation between mindfulness and L2 performance (path b-c), this meta-analysis included the following studies: Alsharhani et al. (2023); Charoensukmongkol (2019); Fathi and Aghamirzaei (2026); Gao (2023); Herman et al. (2023); Ashegh Navaie (2018); Sheikhzadeh and Khatami (2017); Sipahutar (2020); Tung (2025); Ulivia et al. (2022); and Zeilhofer and Sasao (2022). The 11 studies with 14 effect sizes yielded *r* = 0.20 (Fisher's *z* = 0.200), high heterogeneity (*I*^2^ = 85.01%, *p* < 0.001), and a significant positive overall effect (*z* = 4.12, *p* < 0.001) (as shown in Figure 3).

**Figure 4:**
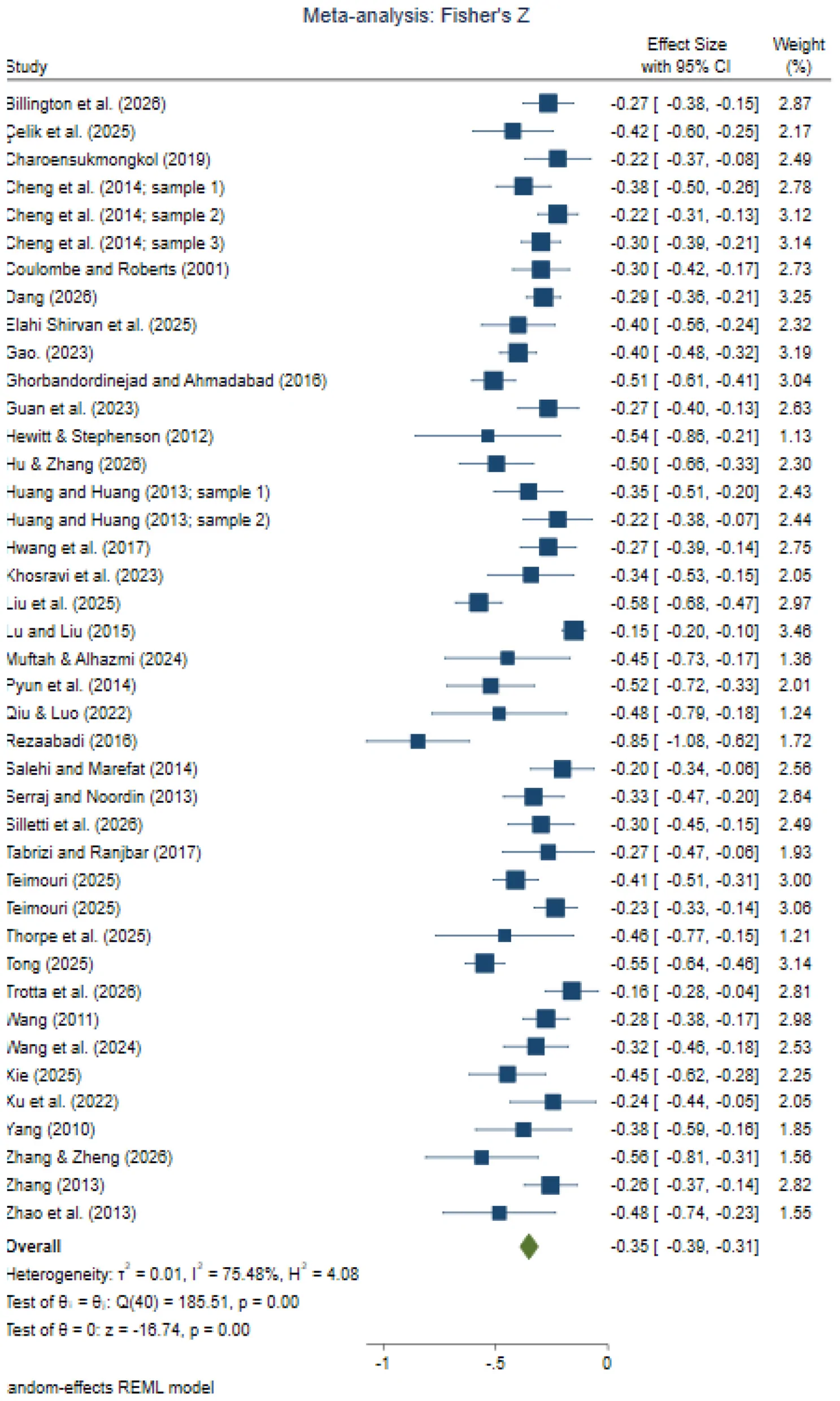
Forest plots of the association between anxiety and L2 performance.

For the direct relation between anxiety and L2 performance (path a-c), this meta-analysis included the following studies: Billington et al. (2026); Çelik et al. (2025); Charoensukmongkol (2019); Cheng et al. (2013); Dang (2026); Elahi Shirvan et al. (2025); Gao (2023); Ghorbandordinejad and Ahmadabad (2016); Guan et al. (2023); Hewitt and Stephenson (2011); Hu and Zhang (2026); Huang and Hung (2013); Hwang et al. (2017); Khosravi et al. (2023); Liu et al. (2025); Lu and Liu (2015); Muftah and Alhazmi (2024); Pyun et al. (2014); Qiu and Luo (2022); Rezaabadi (2016); Salehi and Marefat (2014); (Serraj and Bt. Noordin (2013); Silletti et al. (2026); Tabrizi and Ranjbar 2017); Teimouri (2025); Thorpe et al. (2025); Tong (2025); Trotta et al. (2026); Wang (2011); Wang et al. (2024); Xie (2025); Xu et al. (2022); Yang (2010); ; Zhang and Zheng (2026); Zhang (2013); Zhao et al. (2013). The 35 studies with 41 effect sizes gave r = −0.34, 95% CI: −0.38, −0.30 (Fisher's *z* = −0.35), considerable heterogeneity (*I*^2^ = 79.8%, *p* < 0.001), and a significant negative overall effect (*z* = −16.74, *p* < 0.001) (as shown in Figure 4). After removing duplicate studies across paths, the meta-analysis included a total of 57 unique independent studies.

Table 1 presents the results of three random-effects univariate meta-analyses. For the relationship between **anxiety and mindfulness**, 13 studies with 17 effect sizes yielded a mean correlation of *r* = −0.32 (95% CI: [−0.27, −0.36]), which was statistically significant (*p* < 0.001), with high heterogeneity (*I*^2^ = 78.7%). For the relationship between mindfulness and L2 performance, 11 studies with 14 effect sizes produced a mean correlation of *r* = 0.20 (95% CI: [0.10, 0.28], *p* < 0.001, *I*^2^ = 85.0%). For the relationship between anxiety and L2 performance, 35 studies with 41 effect sizes gave a mean correlation of *r* = −0.34 (95% CI: [−0.38, −0.30], *p* < 0.001, *I*^2^ = 79.8%). All three pooled effect sizes were significant, with substantial heterogeneity across studies. Publication bias was assessed using funnel plots (as shown in supplement Figures 1–3). Egger's tests showed no significant publication bias for the anxiety–mindfulness relation (β*1* = 0.17, SE = 2.964, *z* = 0.06, *p* = 0.953), but significant small-study effects for anxiety–L2 performance (β*1* = −1.85, SE = 0.693, *z* = −2.68, *p* = 0.008) and mindfulness–L2 performance (β*1* = 3.45, SE = 1.544, *z* = 2.23, *p* = 0.026). Trim-and-fill correction, however, altered the pooled estimates only marginally, indicating that the overall meta-analytic conclusions remain robust.

**Table 1:** Results of random-effects univariate meta-analyses.

Relation	*N*	*k*	*r*	95% CI	*p*	*I^2^*
Anxiety → mindfulness	13	17	−0.32	[−0.36, −0.27]	<0.001	78.7%
Mindfulness → L2 performance	11	14	0.20	[0.10, 0.28]	<0.001	85.0%
Anxiety → L2 performance	35	41	−0.34	[−0.38, −0.30]	<0.001	79.8%

*n* = number of studies; *k* = number of effect sizes; SE = standard error; CI = confidence interval.

### 3.3 Description for subgroup analyses

To examine whether the observed effect sizes varied across study–level characteristics, subgroup analyses were conducted for the three bivariate relations. All subgroup results must be interpreted with extreme caution, given the extremely limited number of independent effect sizes (*k*) in several subgroups: only one effect size for adolescents in the mindfulness–L2 performance group, zero eligible Western studies for the mindfulness–L2 relation, and fewer than three effect sizes for other L2 subgroups. Small subgroup *k* leads to unstable pooled correlations and inflated heterogeneity estimates, so no definitive moderating conclusions can be drawn from current subgroup comparisons. The moderators included age (adolescents vs. adults), educational level (*K*-12/school vs. higher education), language type (English vs. other L2), L2 skill domain (comprehensive vs. single skill), and study region (East Asia vs. Western countries). As shown in Table 2, the correlation between anxiety and mindfulness was negative and significant across all subgroups, with slightly larger magnitudes for adolescents (*r* = −0.29) and for studies using other L2 (*r* = −0.39).

**Table 2:** Subgroup analyses for the three bivariate relations.

Moderator	Anxiety–mindfulness					Mindfulness–L2 performance					Anxiety–L2 performance				
	*n*	*k*	r	*p*	*I^2^*	*n*	*k*	r	*p*	*I^2^*	*N*	*k*	r	*p*	*I^2^*
Age															
Adolescents	443	2	−0.29	<0.001	91.4%	31	1	0.26	0.158	0%	2,842	8	−0.34	<0.001	78.2%
Adults	4,148	10	−0.24	<0.001	90.8%	3021	13	0.21	<0.001	90.9%	10,501	27	−0.31	<0.001	83.5%
Education level															
K−12/school	443	2	−0.29	<0.001	91.4%	31	1	0.26	0.158	0%	2,037	6	−0.33	<0.001	72.4%
Higher education	4,148	10	−0.24	<0.001	90.8%	3021	13	0.21	<0.001	90%	11,989	32	−0.32	<0.001	85.1%
Language type															
English	4,449	11	−0.24	<0.001	90.0%	2742	12	0.21	<0.001	90%	13,816	36	−0.32	<0.001	84.0%
Other L2	142	1	−0.39	<0.001	0%	279	2	0.24	<0.001	0%	210	2	−0.34	<0.01	65.3%
L2 skill domain															
Comprehensive	3,736	8	−0.23	<0.001	87.0%	1220	6	0.21	<0.001	87.2%	7,637	17	−0.30	<0.001	82.6%
Single skill	855	4	−0.34	<0.001	71.2%	1832	8	0.22	<0.001	90.8%	6,389	21	−0.34	<0.001	85.7%
Study region															
East Asia	3,449	7	−0.21	<0.001	93.1%	761	2	0.23	<0.001	0%	10,688	26	−0.32	<0.001	83.9%
Western countries	343	3	−0.33	<0.001	0%	N/A	N/A	N/A	N/A	N/A	1,799	7	−0.29	<0.001	75.8%

*n* = total sample size; *k* = number of effect sizes; *r* = weighted mean correlation; *I*^2^ = percentage of total variation due to heterogeneity. N/A = No eligible studies available for this subgroup; For the mindfulness–L2 performance relation, no eligible studies from Western countries were identified. Multiple subgroups contain fewer than 3 independent effect sizes (k <3). Pooled correlation coefficients and I^2^ values from these small–sample subgroups are unstable and exploratory only; no causal or generalizable inferences are permitted.

For the mindfulness–L2 performance relation, the positive correlation was consistently significant in adult, higher education, and English-language subgroups, but the adolescent group (based on a single effect size) did not reach significance (*r* = 0.26, *p* = 0.158). The anxiety–L2 performance relation was negative and significant in every subgroup, with effect sizes ranging from −0.29 (Western countries) to −0.34 (adolescents, single skill). Notably, the number of studies in some subgroups (e.g., adolescents, other L2, Western countries for mindfulness–L2 performance) was small, and the high heterogeneity (*I*^2^ up to 93.1%) in several comparisons warrants cautious interpretation. Overall, no clear pattern of moderation by age or educational level emerged, but the limited sample sizes preclude definitive conclusions.

### 3.4 Meta–analytic structural equation modeling

**Figure 5:**
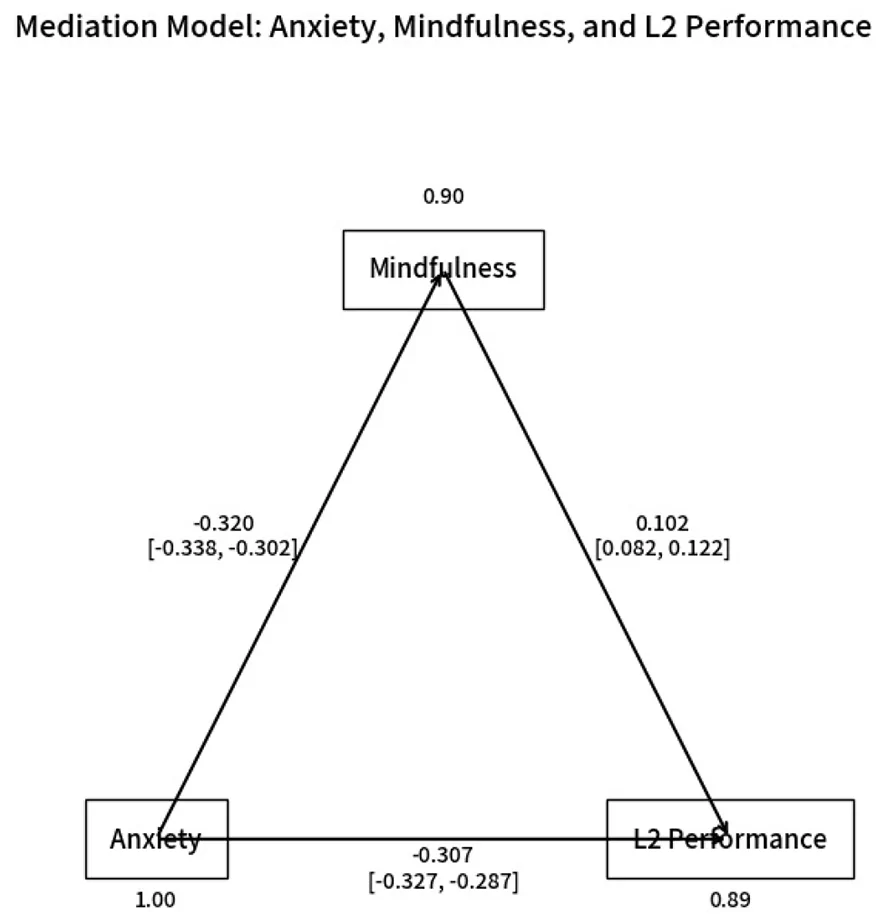
The final mediation model with standardized path coefficients.

Table 3 presents the results of the mediation analysis. The direct path from anxiety to mindfulness was negative and significant (β = −0.320, *p* < 0.001). The path from mindfulness to L2 performance was positive and significant (β = 0.102, *p* < 0.001). The direct effect of anxiety on L2 performance was also negative and significant (β = −0.307, *p* < 0.001). The indirect effect of anxiety on L2 performance through mindfulness was small but significant (β = −0.033, *p* < 0.001), indicating partial mediation. The total effect of anxiety on L2 performance was negative and significant (β = −0.340, *p* < 0.001). The model explained 10.2% of the variance in mindfulness and 11.3% of the variance in L2 performance (as shown in Figure 5). The proposed mediation model was fit to the pooled correlation matrix. All parameters were estimated using full–information maximum likelihood.

**Table 3:** Standardized path coefficients and effect decomposition for the mediation model.

Path/effect	β (std.all)	SE	*z*–value	*p*–value	95% CI
Direct paths					
Anxiety → mindfulness (a)	−0.320	0.009	−33.78	<0.001	[−0.338, −0.302]
Mindfulness → L2 performance (b)	0.102	0.010	10.29	<0.001	[0.089, 0.129]
Anxiety → L2 Performance (c)	−0.307	0.010	−31.14	<0.001	[−0.327, −0.287]
Indirect effect					
Anxiety → mindfulness → L2 (a × b)	−0.033	0.003	−9.843	<0.001	[−0.041, −0.029]
Total effect					
Anxiety → L2 Performance	−0.340	0.009	−36.15	<0.001	[−0.358, −0.302]

N = 10,000. SE = standard error. All coefficients are standardized (std.all). 95% CI computed as estimate ± 1.96 × SE. R^2^ = 0.102 for mindfulness, 0.113 for L2 performance.

## 4 Discussion

### 4.1 Summary of findings

This MASEM study provides robust evidence confirming the widely reported bivariate associations in L2 acquisition research: FLA is significantly negatively correlated with both trait mindfulness and L2 performance, and mindfulness is significantly positively correlated with L2 performance. The most critical and novel finding is that although mindfulness plays a statistically significant mediating role in the FLA–L2 performance relationship, the magnitude of this indirect effect is very small (β = −0.033), accounting for only approximately 10% of the total effect of FLA on L2 performance (direct effect β = −0.307). In meta–analytic convention, standardized indirect effects below 0.08 are generally classified as negligible small effects. This value indicates mindfulness only accounts for roughly 10% of the total negative association between foreign language anxiety and L2 performance. For language educators, this statistical significance cannot translate to meaningful intervention gains: even if learners substantially raise trait mindfulness, the anxiety–induced loss in language proficiency will only be marginally alleviated, rather than substantially offset. One plausible explanation for the weak indirect effect is that mindfulness primarily operates on the affective pathway of FLA: it enhances emotional awareness and non–reactive acceptance, reducing rumination and emotional depletion associated with anxiety, but does not directly address the cognitive–skill pathway of FLA, which includes reduced learning engagement, increased avoidance of language practice, and pre–existing gaps in linguistic knowledge. Given that L2 achievement is also shaped by cognitive abilities, learning motivation, classroom environment, and input quantity–factors largely independent of mindfulness levels–it is understandable that mindfulness contributes only minimally to the anxiety–performance association. It is important to note that this mediation pattern is estimated from synthesized correlations across studies, given that only three primary studies measured FLA, mindfulness, and L2 performance simultaneously.

### 4.2 Theoretical implications

This study extends the classic meta–analysis by Teimouri et al. (2019). The effect size for the anxiety–L2 performance relation (*r* = −0.34) is highly consistent with Teimouri et al's meta–analytic estimate of *r* = −0.33, validating the robustness of our sample and, for the first time, testing a potential mediating mechanism of this well–documented association. Our results align with multiple theoretical frameworks proposed in the literature. First, they are broadly consistent with Attentional Control Theory (Eysenck et al., 2007), which posits that anxiety may contribute to lower executive function and attentional control, linking to reduced performance. The observed negative association between FLA and mindfulness, and positive association between mindfulness and L2 performance, support the proposition that mindfulness may counteract anxiety–related attentional disruption by enhancing attentional control and emotional regulation (Jha et al., 2007). However, it must be noted that because none of the primary studies directly measured attentional control, our results cannot provide direct evidence for this specific mechanism. Second, our findings are consistent with the predictions of Monitor and Acceptance Theory (MAT: Lindsay and Creswell, 2019). The slightly larger effect sizes observed for multi–facet FFMQ measures of mindfulness (vs. single–dimension MAAS measures) suggest that both attentional monitoring and non–reactive acceptance components of mindfulness contribute to buffering anxiety's effects, but the small overall indirect effect indicates that these processes have limited explanatory power for the FLA–L2 association. Contrary to the prevailing assumption in educational psychology and L2 acquisition research that mindfulness serves as a powerful buffer against anxiety–related learning impairment, our data show that the indirect effect of mindfulness explains only about one–tenth of the total association between FLA and L2 performance. These findings suggest that the benefits of trait mindfulness for mitigating anxiety–related L2 underperformance may be more modest than some prior theoretical discussions have implied. Instead, the negative impact of anxiety on L2 learning outcomes is largely produced through broader cognitive and motivational mechanisms that cannot be fully explained by trait mindfulness, such as self–efficacy (Bandura and Wessels, 1997), cognitive reappraisal (Gross and John, 2003), and self–regulation strategies (Zimmerman, 2000). Future research should examine these variables as parallel or serial mediators to provide a more comprehensive account of how anxiety affects language learning.

### 4.3 Practical implications

The findings have cautious practical implications for language educators and curriculum designers. Importantly, the current study only examined cross–sectional associations between trait mindfulness and learning outcomes, so no causal claims about the effectiveness of mindfulness interventions can be made. Given the very small magnitude of the indirect effect, mindfulness–based activities may be considered as a complementary, rather than primary, component of broader strategies to support anxious language learners, and should only be implemented in alignment with existing evidence from randomized controlled trials of mindfulness in educational settings. No reliable evidence of moderation by age or educational level was detected in the current exploratory analyses, suggesting that the associations among FLA, trait mindfulness, and L2 performance may be relatively consistent across learner populations. However, this interpretation is preliminary due to the small number of studies in the adolescent and K−12 subgroups; further research with larger, more diverse samples is needed to confirm whether these relationships hold across all learner groups. The trivial magnitude of the indirect effect further differentiates statistical and practical significance. While the mediation pathway exists statistically, its educational utility is limited. Language instructors should not expect mindfulness practice to produce tangible, large improvements in anxious learners' speaking, listening, reading, or writing performance. Mindfulness training can only serve as auxiliary emotional regulation support rather than a core intervention strategy for mitigating language anxiety deficits.

### 4.4 Limitations and future directions

Several limitations should be acknowledged. First, the meta–analytic estimates in all models exhibited high between–study heterogeneity (*I*^2^ = 78.7–85.0%), indicating substantial variation in effect sizes across primary studies. Potential sources of heterogeneity include differences in measurement instruments for anxiety and mindfulness, differences in the L2 skills measured, differences in learners' sociocultural backgrounds and target language contexts, and differences in study design characteristics. Although we examined the moderating effects of age and educational level, these two variables explained only a very small portion of the observed heterogeneity. The moderator subgroup analyses suffered from uneven and insufficient primary study numbers, especially adolescent/K−12 learner subgroups and non–English L2 subgroups with very few effect sizes. Small subgroup samples may produce biased pooled effect sizes and cannot reliably detect true moderating effects. Future research should conduct further moderator analyses based on larger pools of primary studies to identify other core sources of heterogeneity.

Substantial high heterogeneity (*I*^2^ = 78.7%−85.0%) was detected across all three bivariate meta–analyses. We initially planned continuous meta–regression to explore additional moderators (e.g., publication year, measurement scale type, research design). However, multiple constraints rendered meta–regression unreliable: (1) Most moderators lacked sufficient within–group study counts to produce stable regression coefficients; (2) severe multicollinearity existed between moderators such as educational level and age; (3) partially overlapping datasets violated the independent sampling assumption required for meta–regression. For these methodological reasons, we only conducted categorical subgroup comparisons instead of continuous meta–regression. Second, the use of synthesized correlation matrices in MASEM represents a key limitation. The three pairwise associations were derived from partially overlapping sets of studies-−13 studies contributed to the anxiety–mindfulness correlation, 11 to the mindfulness–L2 performance correlation, and 35 to the anxiety–L2 performance correlation, with only 3 of the 57 included studies measuring all three constructs simultaneously. This partial overlap means that the mediation model is effectively estimated from a synthetic correlation matrix in which different subsets of studies inform different paths. The indirect effect (a × b) is therefore a composite estimate derived from different samples rather than an observed mediation effect within any single study. This aggregation may introduce bias if the studies contributing to the anxiety–mindfulness path systematically differ from those contributing to the mindfulness–L2 performance path (e.g., in terms of sample characteristics, measures, or contexts). Moreover, because the direct and indirect paths are not estimated from the same participants, the decomposition of the total effect into direct and indirect components should be interpreted as an approximation rather than a precise within–person accounting. Future MASEM research should prioritize primary studies that measure anxiety, mindfulness, and L2 performance in the same sample to exhibits positive associations with the validity of mediation estimates. Third, the cross–sectional nature of most included studies precludes strong causal inferences. Although the mediation model implies a directional relationship, we cannot definitively conclude that anxiety causes reduced mindfulness, which in turn causes poorer L2 performance. Alternative causal directions (e.g., poor L2 performance increases anxiety, which correlates negatively with mindfulness) or bidirectional relationships are possible. Future research should employ longitudinal designs with multiple time points to establish temporal precedence and test causal mediation. Fourth, the measures of anxiety, mindfulness, and L2 performance varied across studies, which may have contributed to the observed heterogeneity. Future research should adopt standardized assessment instruments to facilitate cross–study comparisons. Fifth, the sample sizes for moderator analyses were relatively small, particularly for the adolescent and K−12 subgroups. Null findings for these moderators should be interpreted with caution, and future research with larger numbers of studies in these categories is needed to definitively determine whether age and educational level moderate the effects. Finally, although the meta–analysis analyses did not detect substantial publication bias, selective reporting of significant effects cannot be fully ruled out. In particular, the small number of studies examining the mindfulness–L2 performance association (*k* = 11) increases the risk that null findings are underrepresented in the published literature, potentially leading to overestimation of the mindfulness–L2 effect size. Future primary studies should prioritize pre–registration to reduce selective reporting bias. Future research should also (1) examine whether the mediating role of mindfulness varies across specific language skills (e.g., speaking, listening, reading, writing) or learning contexts; (2) test which specific facets of mindfulness (e.g., non–reactivity, acting with awareness) most strongly mediate the anxiety–performance relationship to inform targeted intervention design; (3) conduct randomized controlled trials of mindfulness interventions to determine whether manipulating mindfulness levels can produce meaningful improvements in L2 performance for anxious learners.

## 5 Conclusions

This MASEM study synthesizes existing empirical evidence to validate the core relationships among FLA, trait mindfulness, and L2 performance: FLA is significantly negatively correlated with both mindfulness and L2 performance, mindfulness is significantly positively correlated with L2 performance, and mindfulness plays a significant partial mediating role in the FLA–L2 relationship. These conclusions refer to an estimated mediation pattern at the meta–analytic level and should not be interpreted as evidence of mediation within any single primary study. The central contribution of this study is to clarify the boundary of mindfulness's mediating effect: although statistically significant, the magnitude of this effect is very small, explaining only about 10% of the total effect of FLA on L2 performance. This indicates that mindfulness is only a peripheral pathway through which anxiety affects L2 learning outcomes, and its beneficial contribution to language learning may be more limited than some existing theoretical discussions suggest. Future research should employ longitudinal designs to clarify the causal direction among the three constructs, incorporate multiple mediating variables at the cognitive, motivational, and environmental levels to explore the core mechanisms through which anxiety affects L2 performance, and conduct randomized controlled trials of mindfulness interventions to provide more reliable evidence for language education practice.

## Data Availability

The original contributions presented in the study are included in the article/Supplementary material, further inquiries can be directed to the corresponding author.
