# Supplementary material for: Does mindfulness mediate the relationship between anxiety and second language performance? A meta-analytic structural equation modeling study
Source: Front Psychol. 2026 Aug 14;17:1921078. doi: 10.3389/fpsyg.2026.1921078 (PMC13526469; doi:10.3389/fpsyg.2026.1921078)
Supplement: Supplementary file 1 [file Table_1.docx]

**Search Strategy： Databases searched: PubMed, PsycINFO, Web of Science (WOS), CNKI**

**Search date: May 5, 2026**

**Supplement Table S1. Web of Science (WOS, SCI & SSCI) Search Results**

| No. | Search Query | Results |
| --- | --- | --- |
| **1** | **AB=(anxi*) OR AB=(anxiety)** | **286372** |
| **2** | **AB=(mindful*) OR AB=(mindfulness)** | **79215** |
| **3** | **AB=("second language" OR L2 OR "foreign language") AND AB=(perform* OR achievement OR proficiency)** | **156938** |
| **4** | **#1 AND #2 AND #3** | **2715** |

**Note: WOS was restricted to SCI and SSCI collections; wildcard * was applied for lexical variants.**

**Supplement Table S2. PsycINFO Search Results**

| No. | Search Query | Results |
| --- | --- | --- |
| **1** | **Abstract: anxi* OR Abstract: anxiety AND Abstract: mindful* OR Abstract: mindfulness AND Abstract: ("second language" OR L2 OR "foreign language" OR second-language) AND Abstract: (perform* OR proficiency OR achievement)** | **1826** |

**Note: All retrieval terms were limited to abstract field.**

**Supplement Table S3. PubMed Search Results**

| No. | Search Query (Title/Abstract) | Results |
| --- | --- | --- |
| **1** | **anxi*[Title/Abstract] OR anxiety[Title/Abstract]** | **398659** |
| **2** | **mindful*[Title/Abstract] OR mindfulness[Title/Abstract]** | **47132** |
| **3** | **"second language"[Title/Abstract] OR L2[Title/Abstract] OR "foreign language"[Title/Abstract] AND perform*[Title/Abstract]** | **32967** |
| **4** | **#1 AND #2 AND #3** | **1152** |

**Note: Search terms were confined to title and abstract fields per PubMed retrieval rules.**

**Supplement Table S4. CNKI Search Results**

| No. | Chinese Search Query | Results |
| --- | --- | --- |
| **1** | **(焦虑 + 焦虑情绪) AND (正念 + 正念训练) AND (第二语言 + 二语 + 外语) AND (语言表现 + 学业成绩 + 二语水平)** | **329** |

**Web of Science = 2715; PsycINFO = 1826; PubMed = 1152; CNKI = 329**

**Total preliminary retrieved articles = 2715 + 1826 + 1152 + 329 = 6022**

**Supplement Table S5 Overview of the characteristics of the included studies for anxiety -mindfulness relationship**

| Author (year) | Age | Educational level | Measurement tools | Language | L2 skill | Study region |
| --- | --- | --- | --- | --- | --- | --- |
| Chang, Hutagalung & Chew (2024) | 13–16 years | Secondary school (junior high) | FLCAS, STAI, CAMM | English | Foreign language classroom anxiety | Malaysia |
| Charoensukmongkol (2019) | 19–26 (M=20.98) | University undergraduate | MAAS, PSCAS, | English (ESL) | Speaking (public speaking) | Thailand |
| Choomchaiyo & Varma (2021) | 18–55+ (mixed) | University students & office workers | FFMQ-SF, PI, FMPS, PLDS, FLCAS-SF, GSE, SASA, etc. | English (EFL) | Self-rated foreign language fluency | Thailand |
| Fallah (2016) | 18–31 (M=20.24) | University undergraduate | MAAS, CSES, FLCAS | English (EFL) | Foreign language anxiety | Iran |
| Fallah, Abdolahzadeh & Yaaghobi (2023) | 17–68 (M=33.13) | Language teachers (private institutes) | Mindfulness in Teaching Scale, ERQ, FLTAS | English / Arabic (EFL) | Foreign language teaching anxiety | Iran |
| Gao (2023) | 17–23 (M=19.52) | University undergraduate | MAAS, FLCAS, MBI-EFL | English | Self-rated foreign language proficiency | China |
| Gordani & Sadeghzadeh (2023) | 18–20 (M=18.3) | University undergraduate (freshmen) | FFMQ, PCQ, FLCAS | English (EFL) | Foreign language anxiety | Iran |
| Morgan & Katz (2021) | 18–19 (M=18.51) | University undergraduate | FMI, FLCAS, MEQ | Spanish (foreign language) | Foreign language classroom anxiety | USA |
| Önem (2015) | Typical university age (approx. 18–22) | University undergraduate | STAI | English | Vocabulary learning | Turkey |
| Romadhona, Wibowo & Mulawarman (2022) | Approx. 14–15 (grade 9) | Junior high school (MTs) | FLCAS, etc. | English | Foreign language classroom anxiety | Indonesia |
| Shen (2022) | Unspecified (university students) | University undergraduate | FFMQ-SF, FLCAS | English (EFL) | Foreign language anxiety | China |
| Tasan, Mede & Sadeghi (2021) | Unspecified (sophomores) | University undergraduate | ELLAS, FLTAS | English | Listening comprehension, reading comprehension | Turkey |

**Supplement Table S6 Overview of the characteristics of the included studies for mindfulness- L2 performance**

| Author (year) | Age | Educational level | Measurement tools | Language | L2 skill | Study region |
| --- | --- | --- | --- | --- | --- | --- |
| Alsharhani, Ghonsooly & Meidani (2023) | 20–28 years | University students | Reflective Thinking Questionnaire, FLRAS, | English (EFL) | Reading comprehension | Iraq |
| Navaie (2018) | 18–35 years | English language institute | IELTS reading test | English (EFL) | Reading comprehension | Iran |
| Charoensukmongkol (2019) | 19–26 (M=20.98) | University undergraduate | MAAS, , PSCAS, | English (ESL) | Speaking (public speaking) | Thailand |
| Fathi & Aghamirzaei (2026) | 19–33 (M=22.04) | University undergraduate (adult EFL learners) | MAAS, SRlang scale, Motivated | English (EFL) | L2 achievement (IELTS mock) | Iran |
| Gao (2023) | 17–23 (M=19.52) | University undergraduate | MAAS, short-form FLCAS, MBI-EFL | English | Self-rated foreign language proficiency | China |
| Sipahutar (2020) | Unspecified (university students) | University undergraduate (English Education) | FFMQ, FLCAS, speaking test | English (EFL) | Speaking performance | Indonesia |
| Tung (2025) | Unspecified (adults, working age) | Adult learners (Learn Welsh Cardiff courses) | language diaries | Welsh (minoritised language) | Language learning motivation (not a specific skill) | UK (Wales, Cardiff) |
| Zeilhofer & Sasao (2022) | 18.78 years (SD = 0.77) | University (first‑year students) | FFMQ | German (L3) | Vocabulary (short‑term retention) | Japan |
| Ulivia, Petrus & Suganda (2022) | 18–22 years | University undergraduate (English Education) | FFMQ, AMS, GPA | English (EFL) | Academic performance (GPA) | Indonesia |

**Supplement Table S7 Overview of the characteristics of the included studies for anxiety -L2 performance relationship**

| Author(s) (year) | Age | Education Level | Measurement Tools | Language | L2 Skill | Study Region |
| --- | --- | --- | --- | --- | --- | --- |
| Celik et al. (2025) | 14–17 years | High school | ELSA, ELPAS, NASA-TLX | English | Speaking | Turkey |
| Charoensukmongkol (2019) | 19–26 years | University | MAAS, State mindfulness | English | Public speaking | Thailand |
| Cheng et al. (2014) | 18–24 years | University | FLCAS, Test anxiety | English | General | China, Canada |
| Dang (2026) | 18–20 | University | Reading anxiety scale | English | Reading | Vietnam |
| Elahi Shirvan et al. (2025) | 22 years | University | FLCAS | English | Conversational | Iran |
| Gao (2023) | 18–35 years | University | MAAS, FLCAS | English | Proficiency | China |
| Ghorbandordinejad & Ahmadabad (2016) | 17–18 | High school | FLCAS | English | Achievement | Iran |
| Guan et al. (2023) | 19–22 years | University | Writing anxiety, MAAS | English | Writing | China |
| Hewit & Stephenson (2012) | Not reported | University | FLCAS | English | General | USA |
| Hu & Zhang (2026) | 18–22 years | University | FLCAS, MAAS | English | General | China |
| Huang & Huang (2013) | 18–30 years | University | STAI, speaking test | English | Speaking | Taiwan |
| Hwang et al. (2017) | 18–22 years | University | FLCAS | English | General | Korea |
| Khosravi et al. (2023) | 17–24 years | Institute | SLWAI, PET | English | Writing | Iran |
| Liu et al. (2025) | 18–22 years | University | FLCAS, MAAS | English | General | China |
| Lu & Liu (2015) | 18–22 years | University | Reading anxiety | English | Reading | China |
| Mufiah & Alhazmi (2024) | 20–23 years | University | Idiodynamic, speaking task | English | Speaking | Saudi Arabia |
| Pyun et al. (2014) | 18–22 years | University | FLCAS | English | General | Korea |
| Qiu & Luo (2022) | 21–28 years | Language school | FLLAS, IELTS | English | Listening | China |
| Rezaabadi (2016) | 16–28 years | IELTS prep | FLLAS, IELTS | English | Listening | Iran |
| Romadhona et al. (2022) | 15 years | Junior high | MBCT, FLCAS | English | Classroom | Indonesia |
| Salehi & Marefat (2014) | 18–22 years | University | FLCAS | English | General | Iran |
| Serraj & Nordin (2013) | Not reported | University | Listening anxiety | English | Listening | Iran |
| Silletti et al. (2026) | 7–8 years | Primary | AEQES, vocabulary test | English | Vocabulary | Italy |
| Tabrizi & Ranjbar (2017) | 15–20 years | High school | Listening anxiety | English | Listening | Iran |
| Teimouri (2025) | 18–25 years | University | FLCAS | English | General | International |
| Thorpe et al. (2025) | 18–41 years | Institute | FLCAS, STAI, PRAAT | English | Speaking | USA |
| Tong (2025) | 18–21 years | University | AEQ, self-efficacy | English | General | China (Wuhan) |
| Trotta et al. (2026) | 7–8 years | Primary | AEQES, vocabulary | English | Vocabulary | Italy |
| Wang (2011) | 18–22 years | University | FLCAS | English | General | China |
| Wang et al. (2024) | 17 years | High school | Writing anxiety | English | Writing | China |
| Xie (2025) | 18–35 years | University | SLWAI, MAILL | Chinese | Writing | China |
| Xu et al. (2022) | 19 years | University | Chinese anxiety | Chinese | Vocabulary | Thailand |
| Yang (2010) | 18–22 years | University | FLCAS | English | General | China |
| Zhang (2013) | 18–19 years | University | FLLAS, IELTS | English | Listening | China |
| Zhang & Zheng (2026) | 18–22 years | University | FLCAS, MAAS | English | General | China |
| Zhao et al. (2013) | 18–22 years | University | FLCAS | English | General | China |

Note. AEQ = Academic Emotions Questionnaire; AEQES = Anxiety in English Class for Elementary Students; AMS = Academic Motivation Scale; CAMM = Child and Adolescent Mindfulness Measure; CSES = Communication Self-Efficacy Scale; EFL = English as a Foreign Language; ELLAS = English Language Listening Anxiety Scale; ELPAS = English Language Performance Anxiety Scale; ELSA = English Language Speaking Anxiety; ERQ = Emotion Regulation Questionnaire; ESL = English as a Second Language; FFMQ = Five Facet Mindfulness Questionnaire; FLCAS = Foreign Language Classroom Anxiety Scale; FLLAS = Foreign Language Listening Anxiety Scale; FLRAS = Foreign Language Reading Anxiety Scale; FLTAS = Foreign Language Teaching Anxiety Scale; FMI = Freiburg Mindfulness Inventory; FMPS = Frost Multidimensional Perfectionism Scale; GSE = General Self-Efficacy Scale; IELTS = International English Language Testing System; L2 = Second Language; MAAS = Mindful Attention Awareness Scale; MAILL = Measure of Anxiety in Language Learning; MBI-EFL = Maslach Burnout Inventory – English as a Foreign Language version; MEQ = Mindfulness Education Questionnaire; NASA-TLX = NASA Task Load Index; OPT = Oxford Placement Test; PLDS = Perceived Listening Difficulty Scale; PSCAS = Public Speaking Class Anxiety Scale; SLWAI = Second Language Writing Anxiety Inventory; SRlang scale = Self-Regulation in language learning scale; STAI = State-Trait Anxiety Inventory; TOEFL = Test of English as a Foreign Language.

Figure 1: Publication Bias Chart **for anxiety -mindfulness relationship**

Figure 2: Publication Bias Chart **for mindfulness- L2 performance**

Figure 3: Publication Bias Chart **for anxiety- L2 performance**

**Supplement Table S8 Summary of the Quality Assessment Table (NOS Scores)**

| **First Author (Year)** | **1. Representativeness** | **2. Selection non-exposed** | **3. Ascertainment exposure** | **4. Outcome not present at baseline** | **5. Comparability (design)** | **6. Additional comparability** | **7. Outcome assessment** | **8. Follow-up duration** | **9. Follow-up completeness** | **Total NOS** | **Quality Grade** |
| --- | --- | --- | --- | --- | --- | --- | --- | --- | --- | --- | --- |
| **Alsharhani et al. (2023)** | 1 | 1 | 1 | 0 | 1 | 1 | 1 | 0 | 0 | **6** | **Moderate** |
| **Billington et al. (2026)** | 1 | 1 | 1 | 0 | 1 | 0 | 1 | 0 | 0 | **5** | **Moderate** |
| **Celik et al. (2025)** | 1 | 1 | 1 | 0 | 1 | 0 | 1 | 0 | 0 | **5** | **Moderate** |
| **Charoensukmongkol (2019)** | 1 | 1 | 1 | 0 | 1 | 1 | 1 | 0 | 0 | **6** | **Moderate** |
| **Cheng et al. (2014)** | 1 | 1 | 1 | 0 | 1 | 1 | 1 | 0 | 0 | **6** | **Moderate** |
| **Choomchaiyo & Varma (2021)** | 1 | 1 | 1 | 0 | 1 | 1 | 1 | 0 | 0 | **6** | **Moderate** |
| **Coulombe & Roberts (2001)** | 1 | 1 | 1 | 0 | 0 | 0 | 1 | 0 | 0 | **4** | **Low** |
| **Dang** | 1 | 1 | 1 | 0 | 1 | 1 | 1 | 0 | 0 | **6** | **Moderate** |
| **Elahi Shirvan et al. (2025)** | 1 | 1 | 1 | 0 | 1 | 0 | 1 | 0 | 0 | **5** | **Moderate** |
| **Fallah (2017)** | 1 | 1 | 1 | 0 | 1 | 1 | 1 | 0 | 0 | **6** | **Moderate** |
| **Fallah et al. (2023)** | 1 | 1 | 1 | 0 | 1 | 1 | 1 | 0 | 0 | **6** | **Moderate** |
| **Fathi & Aghamirzaei (2025)** | 1 | 1 | 1 | 0 | 1 | 1 | 1 | 0 | 0 | **6** | **Moderate** |
| **Gao (2023)** | 1 | 1 | 1 | 0 | 1 | 1 | 1 | 0 | 0 | **6** | **Moderate** |
| **Ghorbandordinejad & Ahmadabad (2016)** | 1 | 1 | 1 | 0 | 1 | 0 | 1 | 0 | 0 | **5** | **Moderate** |
| **Gordani & Sadeghzadeh (2023)** | 1 | 1 | 1 | 0 | 1 | 1 | 1 | 0 | 0 | **6** | **Moderate** |
| **Guan et al. (2023)** | 1 | 1 | 1 | 1 | 1 | 0 | 1 | 1 | 1 | **8** | **High** |
| **Herman et al. (2023)** | 1 | 0 | 1 | 0 | 0 | 0 | 1 | 0 | 0 | **3** | **Low** |
| **Herman et al. (2024)** | 1 | 1 | 1 | 0 | 1 | 0 | 1 | 0 | 0 | **5** | **Moderate** |
| **Hewitt & Stephenson (2011)** | 1 | 1 | 1 | 0 | 0 | 0 | 1 | 0 | 0 | **4** | **Low** |
| **Hu & Zhang (2026)** | 1 | 1 | 1 | 1 | 1 | 0 | 1 | 1 | 1 | **8** | **High** |
| **Huang & Hung** | 1 | 1 | 1 | 0 | 1 | 0 | 1 | 0 | 0 | **5** | **Moderate** |
| **Hwang et al. (2017)** | 1 | 1 | 1 | 1 | 1 | 0 | 1 | 1 | 1 | **8** | **High** |
| **Khosravi et al. (2023)** | 1 | 1 | 1 | 1 | 1 | 0 | 1 | 1 | 1 | **8** | **High** |
| **Liu et al. (2025)** | 1 | 1 | 1 | 0 | 1 | 1 | 1 | 0 | 0 | **6** | **Moderate** |
| **Lu & Liu (2015)** | 1 | 1 | 1 | 0 | 1 | 1 | 1 | 0 | 0 | **6** | **Moderate** |
| **Morgan & Katz (2021)*** | 1 | 1 | 1 | 1 | 1 | 1 | 1 | 1 | 1 | **9** | **High** |
| **Muftah & Alhazmi (2024)** | 0 | 0 | 1 | 0 | 0 | 0 | 1 | 0 | 0 | **2** | **Low** |
| **Navaie (2018)** | 1 | 1 | 1 | 1 | 1 | 0 | 1 | 1 | 1 | **8** | **High** |
| **Pyun et al. (2014)** | 1 | 1 | 1 | 0 | 1 | 0 | 1 | 0 | 0 | **5** | **Moderate** |
| **Qiu & Luo (2022)** | 1 | 1 | 1 | 1 | 1 | 0 | 1 | 1 | 1 | **8** | **High** |
| **Rezaabadi (2016)** | 1 | 1 | 1 | 1 | 0 | 0 | 1 | 1 | 1 | **7** | **High** |
| **Romadhona et al. (2022)** | 1 | 1 | 1 | 1 | 1 | 1 | 1 | 1 | 1 | **9** | **High** |
| **Salehi & Marefat (2014)** | 1 | 1 | 1 | 0 | 1 | 0 | 1 | 0 | 0 | **5** | **Moderate** |
| **Serraj & Noordin (2013)** | 1 | 1 | 1 | 0 | 1 | 0 | 1 | 0 | 0 | **5** | **Moderate** |
| **Sheikhzadeh & Khatami (2017)** | 1 | 1 | 1 | 0 | 1 | 0 | 1 | 0 | 0 | **5** | **Moderate** |
| **Shen (2022)** | 1 | 1 | 1 | 0 | 1 | 0 | 1 | 0 | 0 | **5** | **Moderate** |
| **Silletti et al. (2026)** | 1 | 1 | 1 | 0 | 1 | 1 | 1 | 1 | 1 | **8** | **High** |
| **Sipahutar (2020)** | 1 | 1 | 1 | 0 | 1 | 1 | 1 | 0 | 0 | **6** | **Moderate** |
| **Tabrizi & Ranjbar** | 1 | 1 | 1 | 1 | 1 | 0 | 1 | 1 | 1 | **8** | **High** |
| **Tasan et al. (2021)** | 1 | 1 | 1 | 1 | 1 | 1 | 1 | 1 | 1 | **9** | **High** |
| **Teimouri (2025)** | 1 | 1 | 1 | 0 | 1 | 1 | 1 | 0 | 0 | **6** | **Moderate** |
| **Thorpe et al. (2025)** | 1 | 1 | 1 | 0 | 1 | 0 | 1 | 1 | 1 | **7** | **High** |
| **Tong (2025)** | 1 | 1 | 1 | 0 | 1 | 0 | 1 | 0 | 0 | **5** | **Moderate** |
| **Trotta et al. (2026)** | 1 | 1 | 1 | 0 | 1 | 1 | 1 | 1 | 1 | **8** | **High** |
| **Tung (2025)** | 1 | 0 | 1 | 0 | 0 | 0 | 1 | 0 | 0 | **3** | **Low** |
| **Ulivia et al. (2022)** | 1 | 1 | 1 | 0 | 1 | 1 | 1 | 0 | 0 | **6** | **Moderate** |
| **Wang (2011)** | 1 | 1 | 1 | 0 | 1 | 0 | 1 | 0 | 0 | **5** | **Moderate** |
| **Wang et al. (2024)** | 1 | 1 | 1 | 0 | 1 | 0 | 1 | 0 | 0 | **5** | **Moderate** |
| **Xie (2025)** | 1 | 1 | 1 | 0 | 0 | 0 | 1 | 1 | 1 | **6** | **Moderate** |
| **Xu et al. (2022)** | 1 | 1 | 1 | 0 | 1 | 0 | 1 | 0 | 0 | **5** | **Moderate** |
| **Yang (2010)** | 1 | 1 | 1 | 0 | 1 | 0 | 1 | 0 | 0 | **5** | **Moderate** |
| **Zeilhofer & Sasao (2022)** | 1 | 1 | 1 | 0 | 1 | 1 | 1 | 0 | 0 | **6** | **Moderate** |
| **Zhang & Saisai (2025)** | 0 | 1 | 1 | 0 | 0 | 0 | 1 | 0 | 0 | **3** | **Low** |
| **Zhang (2013)** | 1 | 1 | 1 | 0 | 1 | 1 | 1 | 0 | 0 | **6** | **Moderate** |
| **Zhao et al. (2013)** | 1 | 1 | 1 | 0 | 1 | 0 | 1 | 0 | 0 | **5** | **Moderate** |
| **Önem (2015)** | 1 | 1 | 1 | 1 | 1 | 1 | 1 | 0 | 0 | **7** | **High** |
